# Bimini Islands: a characterization of the two major nursery areas; status and perspectives

**DOI:** 10.1186/2193-1801-3-270

**Published:** 2014-05-28

**Authors:** Claudia Trave, Marcus Sheaves

**Affiliations:** School of Marine and Tropical Biology, James Cook University, Townsville, QLD 4811 Australia; Centre for Tropical Water & Aquatic Ecosystem Research (TropWATER), James Cook University, Townsville, QLD 4811 Australia

## Abstract

Bimini Islands (Bahamas, 25°44′ N 79°16′ W) are characterized by a unique tropical marine environment which provides critical nursery habitats and food resources for many important species of ecological and economical value. Two areas are particularly important in the function and dynamics of the local marine environment: North Sound and South Bimini. Since 1998 the northern part of the island has been subject to an intense urbanization process that involves the construction of an extensive touristic complex. Over the years this activity has radically modified a substantial portion of the land, and part of the underwater environment as well, threatening the fragile balance of the North Sound nursery ground. Effects on marine habitats and on local species have been reported, and although some measures to limit the damage have already been taken, the local ecosystem could ultimately suffer from continuation of the construction work on the area. In 2010, we performed surveys of both main nursery grounds to assess the current ecological status and the main differences between the two areas, investigating macrobenthic epifauna abundance, seagrass density and abiotic parameters. The results of this study indicate that the ecosystem still appears in reasonably healthy condition, although showing some concerning trends. These data provide baseline conditions to assess further changes, and possibly to support the development of plans for the conservation of the North Sound and South Bimini coastal ecosystems.

## Introduction

Bimini comprises two small subtropical islands (North and South Bimini) located in the North West corner of the archipelago of the Commonwealth of the Bahamas. The islands are arranged in a triangle enclosing a central lagoon of approximately 21 km^2^ (Morrisey and Gruber 1993; Voss and Voss 1960). Its geographic position on the western edge of the Great Bahama Bank facing the Florida Strait, together with its geomorphologic, hydrologic and climatic characteristics, allowed the development of a diversity of marine biotopes such as coral reefs, seagrass beds, mangrove forests, sand flats and banks (Bell et al. 2006; Hedgpeth 1957; Marbà et al. 1994; Newman et al 2007; Scoffin 1970; Turekian 1957). The presence of such a diverse marine ecosystem, with complex and interconnected habitats, makes this an area of rich biodiversity with abundant resources and a relatively pristine environment (Rönnback 1999; Stoner 1980). The health of these diverse ecosystems is vital to the livelihoods of the local population, either directly through commercial and recreational fishing or indirectly via tourism, Bimini’s main source of economic sustainability.

Bimini’s abundant mangrove forests and seagrass meadows play a fundamental role in maintaining local biodiversity (Jennings et al. 2008). These two habitats are a source of primary production, offering foraging opportunities to many fish and invertebrates, and providing critical nursery grounds for benthic and nektonic species of ecological and economic value. For instance, Bimini’s shallow water ecosystems are inhabited by *Negaprion brevirostris* (commonly known as lemon shark), considered by the World Conservation Union/Species Survival Commission (IUCN/SSC) as a ‘near-threatened’ species (Jennings et al. 2008; Murchie et al. 2010); *Pristis pectinata*, the smalltooth sawfish listed as an endangered species by the Convention on International Trade in Endangered Species of Wild Fauna and Flora (CITES) since 2007 (Feldheim et al. 2010), and *Albula vulpes* (Morrisey and Gruber 1993; Newman et al. 2007) the target of Bimini’s world renown bonefish sport fishing industry. These species, and many more, depend on the presence and well-being of mangroves and seagrass meadows for their survival, particularly in the early stages of their life. Mangrove roots form an intricate web that offers protection and shelter to juvenile nektonic organisms from larger predators (Nagelkerken et al. 2002; Parrish 1989). Seagrass meadows perform a similar function for benthic organisms while also providing rich grazing and hunting grounds for many fish species (Nagelkerken et al. 2002; Parrish 1989). Once adulthood is reached, most fish move to deeper waters and progress through a series of different habitats, in some cases migrating to the open ocean or even to other islands (Kenneth 2005). The condition of mangrove forests and seagrass meadows along the coastline is therefore of utmost importance for the preservation of Bimini’s marine ecosystem as well as supporting the biodiversity of neighbouring islands.

In 1998, work on the construction of a vast tourist complex known as “Bimini Bay Resort and Marina” began in the northern part of the island close to the North Sound, one of two of Bimini’s nursery grounds (Bimini Bay Project report 2008). Work consisted primarily of site clearing, mangrove cutting and channel dredging, and resulted in progressive modifications of habitats, both on land and underwater, leading to notable effects on local marine habitats (Hussey 2003; Jennings et al. 2008, 2012; Newman et al. 2007; Sealey 2004).

As of 2014, work is still being conducted and has reached the northern tip of the North island. In order to protect and preserve the North Sound ecosystem, a marine protected area was established in 2000 by the Bahamian Government to include the Eastern half of the sound (both land and underwater). However, despite these conservation measures there are concerns that the local benthic invertebrates and fish population could suffer from the substantial reduction and alteration of their natural habitats (Jennings et al. 2008, Bimini Biological Field Station personal communication).

Understanding and monitoring the direction of change is critical for the long-term preservation of Bimini’s near-shore ecosystems, because determining the current status of the ecosystem, particularly relative to lower trophic levels, will provide a reference standard against which future alterations may be judged. Consequently, we conducted a survey in order to establish a baseline characterization of the subtidal benthic ecosystems of Bimini’s main shallow water fish nursery grounds and determine any major ecological differences existing between or within such areas.

The study investigated the benthic ecosystems of both North Sound and South Bimini, areas similar in ecological structure but with contrasting exposure to the open ocean and to anthropogenic activities, through the assessment of macrobenthic epifaunal abundance, seagrass density and abiotic parameters.

## Materials and methods

### Study areas and sites

**Figure 1:**
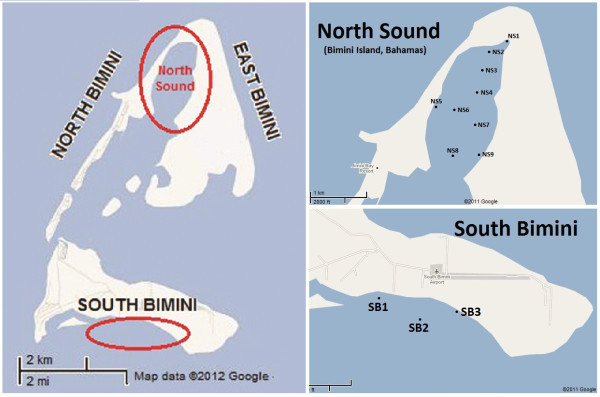
**Map of study areas and site disposition.**

**Figure 2:**
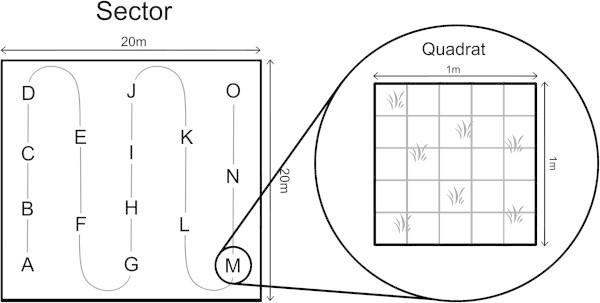
**Example of study sectors and quadrats disposition.**

The characterization of the habitats was performed for the two nursery grounds present in Bimini Islands: the North Sound (9 sites across 2.5 km^2^) and South Bimini (3 sites across 1.45 km^2^) (Figure 1). All sites were chosen from a pre-existing list based on a ISODATA unsupervised habitat classification (Hussey 2003). As sites in the 2003 study were chosen for being representative for all different benthic habitats, their distribution in the North Sound resulted uneven, with only one located on the western side of the sound. Each individual site was marked by a set of coordinates using Wide Area Augmentation System - Global Positioning System (Garmin Inc.), and identified by the specific acronyms, as shown in Figure 1.Four transects (identified as sectors 1, 2, 3 and 4), each defined as a square area of 20 × 20 meters, were placed at random within each of the 12 sites to ensure representative coverage. Quantitative measurements were performed in one meter square quadrats marked with the letters A through O and arranged in a snake-like pattern inside each sector, for a total of 60 quadrats per site (Figure 2).

In order to minimize the impact of the survey on the studied habitats, particular attention was given while performing the measurements as to avoid altering the bottom structure or damaging the local flora and fauna.

The same data gathering and survey methodology used by Hussey (2003) have been utilized in this study, in order to make any comparison or reference as meaningful as possible.

### Abiotic parameters

Measurements of abiotic parameters were carried out at the beginning of each sector survey to minimize the possibility of errors caused by the researcher’s movements in the water and/or silt disturbance, particularly during low tide or in shallow water areas. Parameters included:

*Salinity*, recorded using an optical refractometer (SPER Scientific 300011), *Underwater horizontal transparency*, measured using a Secchi disk (Preisendorfer 1986) and representing the maximum horizontal distance (cm) the disk was visible underwater, *Sediment depth*, measured as depth to pavement in cm, evaluated by pushing a metal rebar vertically through the sand/silt until it contacted the hard rock beneath.

Sample size was n = 4/site in both areas for salinity and visibility, and n = 300/site for sediment depth (5 measures × 15 quadrats × 4 sectors).

Seasonal variations were taken into account when analyzing the data and, although North Sound salinity was recorded predominantly during the wet season, while in South Bimini data were collected closer to the dry season, the input of freshwater from the few thunderstorms was not considered a major factor in the differences observed between the two areas and within the North Sound. Measurements were conducted between high and low tide, to take advantage of slack water periods as much as possible, and only when weather conditions were deemed good and tidal changes would have quickly taken care of the temporary freshwater input by diluting it.

### Analysis of biota

Macrobenthic organisms were identified and their abundance measured at every site by counting the number of individuals/m^2^. Seagrass density was characterized non-destructively by counting the number of blades.

Because seagrass meadows represent the main underwater habitat in both nursery areas and seagrass occurrence and condition are primary indicators of ecosystem health (Orth et al. 2006), additional measurements were carried out in order to determine the status of the two main species present: *Thalassia testudinum* and *Halodule wrightii*.

Blade length and organic biomass were calculated using live blades of seagrass collected for both species by cutting at the junction between the leaves and the plant’s short shoot. A sample of thirty blades per species was collected in each site (as per Hussey 2003). The blades were manually cleaned of sediment and epiphytic organisms, measured (length in cm) and sun dried to obtain their cumulative dry weight (g).

### Statistical analysis

Each of the abiotic parameters was compared individually between North Sound and South Bimini using t-tests. The overall abiotic characters of the different sites were compared using Principal Components Analysis (PCA) on normalized data using PRIMER-R. Biological assemblage structures for North Sound and South Bimini were compared using Multidimensional Scaling (MDS) performed on log(x + 1) transformed density data for the ten most commonly occurring species recorded in the North Sound and South Bimini sites based on Bray-Curtis similarities.

## Results

### Abiotic parameters

The results of the comparative analysis between the North Sound and South Bimini for the abiotic parameters – water salinity, visibility and sediment thickness – observed in this study are summarized in Table 1.

**Table 1:** **Average values for abiotic parameters in North Sound and South Bimini**

Parameter	North Sound Mean ± SD	South Bimini Mean ± SD	p value
Water salinity (ppt)	38.06 ± 3.27 (n = 36)	40.00 ± 1.65 (n = 12)	< 0.001
Underwater visibility (cm)	418.93 ± 167.67 (n = 36)	731.17 ± 152.83 (n = 12)	< 0.0001
Sediment depth (cm)	57.86 ± 32.97 (n = 2700)	62.11 ± 32.13 (n = 900)	< 0.001

**Figure 3:**
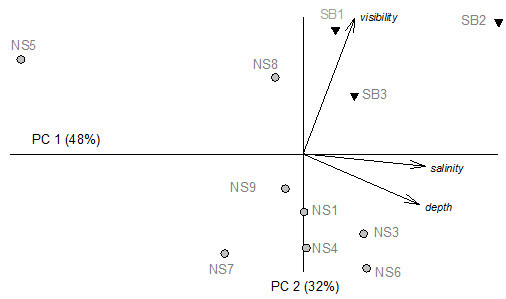
**Principal Components Analysis (PCA) on normalised data for the abiotic parameters.** Grey circles = North Sound, black triangles = South Bimini. The longest vector (visibility) represents a correlation of r = 0.925 with the ordination space.

Underwater visibility differed significantly between the two study areas, with mean values in South Bimini 1.75-fold higher than in North Sound (Table 1). Although mean salinity was similar in the two areas it was much more variable at North Sound with a variance to mean ratio (VMR) (0.281) four times as large as that at South Bimini (0.068).Based on the PCA, water visibility was the main discriminating factor between the North Sound and South Bimini values, while water salinity and sediment thickness seem to account for the high variability among sites belonging to the same area (Figure 3).Abiotic parameters varied among the different sites in the North Sound particularly between the eastern part of the lagoon (pristine) and the western side (close to construction site), with NS8 and NS5 showing the most extreme values (Figure 3). On the other hand, the three South Bimini sites showed an overall fairly uniform profile when considering the mean values recorded for water visibility and salinity, while a high variability was observed in sediment thickness.

### Macrobenthic epifauna

Overall, 47 macrobenthic species were identified in the two areas, including 23 species of seagrass, algae, sponges, anemone and corals (Tables 2 and 3).

**Table 2:** **List of botanical species identified in North Sound and South Bimini habitats**

Flora		
Phylum	Class	Species
Tracheophyta	Angiospermae	**Seagrass**
		*Halodule wrightii*
		*Thalassia testudinum*
		**Mangroves**
		*Rhizophora mangle*
Chlorophyta	Chlorophyceae	**Green algae**
		*Acetabularia spp.*
		*Anadyomene stellata*
		*Batophora oerstedii*
		*Cladophora spp.*
		*Halimeda spp.*
		*Penicillus spp.*
		*Rhiphocephalus phoenix*
		*Udotea spp.*
		*Valonia macrophysa*
Rhodophyta	Rhodophyceae	**Red algae**
		*Ceramium spp.*
		*Laurencia spp.*

**Table 3:** **List of zoological species identified in North Sound and South Bimini habitats**

Fauna		
Phylum	Class	Species
Porifera	Demospongiae	**Sponges**
		*Cliona vastifica*
		*Aplysina cauliformis*
		*Chondrilla nucula*
		*Dysidea etheria*
		*Hyrtios violaceus*
		*Ircinia strobilina*
		*Pellina carbonaria*
Cnidaria	Anthozoa	**Corals**
		*Porites furcata*
		*Siderastrea radians*
		**Anemone**
		*Viatrix globulifera*
	Scyphozoa	**Jellyfish**
		*Cassiopea spp.*
Anellida	Polichaeta	**Worms**
		*Arenicola cristata*
		*Eupolymnia crassicornis*
Arthropoda	Malacostraca	**Crustaceans**
		*Clibanarius spp.*
		*Lysiosquilla scabricauda*
		*Penaeid spp.*
		*Portunus pelagicus*
Mollusca	Gastropoda	**Shells**
		*Batillaria minima*
		*Calliostoma yucatecanum*
		*Cerithium atratum*
		*Cerithium muscarum*
		*Fasciolaria tulipa*
		*Prunum apicinum*
		*Nassarius albus*
		*Polinices lacteus*
		*Strombus gigas*
	Bivalvia	*Atrina spp.*
		*Chione spp.*
		*Divaricella quadrisulcata*
		*Pteria colymbus*
Echinodermata	Echinoidea	**Sea urchins and sea stars**
		*Lytechinus variegatus*
		*Oreaster reticulatus*
Chordata	Ascidiacea	**Tunicate**
		*Ecteinascidia turbinata*

**Figure 4:**
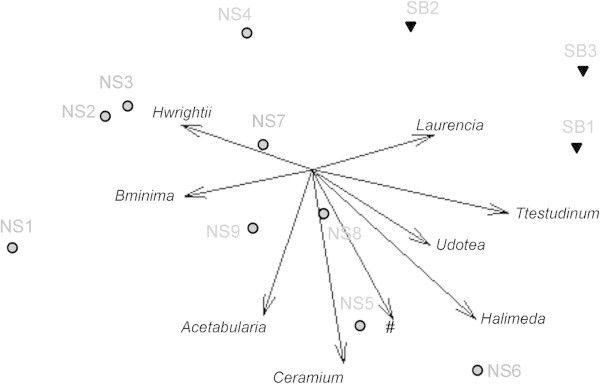
**Multidimensional Scaling (MDS) performed on density data for the most commonly occurring taxa.** Grey circles = North Sound, black triangles = South Bimini. # this vector represents 9 highly correlated taxa: *Acauliformis*, *Boerstedii*, *Cassiopea*, *Cnucula*, *Cyucatecanum*, *Detheria*, *Ftulipa*, *Hviolaceus*, and *Penicillus*. The longest vector (*Halimeda*) represents a correlation of r = 0.815 with the ordination space. Stress = 0.07.

Of these 47 species, ten were common and abundant in the two study areas, while all other species were only found occasionally, sometimes even being recorded only once in the whole survey. The density and analysis of the ten most common benthic species identified in the two areas are summarized in Table 4 and Figure 4.

**Table 4:** **Density of the most frequent and abundant benthic species in North Sound and South Bimini**

Species	North Sound Mean* ± SD	South Bimini Mean* ± SD
*Acetabularia spp.*	4.14 ± 15.73	0.08 ± 0.68
*Batillaria minima*	22.31 ± 65.97	0.02 ± 0.13
*Batophora oerstedii*	186.68 ± 311.16	155.02 ± 195.57
*Ceramium spp.*	2.86 ± 10.21	n.p.
*Ecteinascidia turbinata*	1.36 ± 6.13	n.p.
*Halimeda spp.*	3.41 ± 7.90	4.43 ± 6.34
*Halodule wrightii*	39.20 ± 51.01	0.98 ± 5.89
*Laurencia spp.*	0.84 ± 3.5	7.74 ± 21.18
*Penicillus spp.*	2.6 ± 6.77	1.62 ± 3.08
*Thalassia testudinum*	36.45 ± 55.38	93.34 ± 60.35

Note. n.p.: not present.

*Mean individuals-blades/m^2^.

There was considerable inter-site variability in biotic assemblages between the two study areas (Table 4, Figure 4), possibly related to the bottom characteristics, abiotic parameters and low (NS1-4 and SB2) versus high exposure (NS5-9, SB1 and SB3) to tidal currents. Some species appeared to be widely distributed in both the North Sound and South Bimini areas, although at varying densities, such as the green algae *B. oerstedii*, and *Penicillus spp*. and the seagrass *T. testudinum* (Table 4). On the other hand, the remainder of the species identified appeared to be present primarily or almost exclusively in one of the two study areas. *Acetabularia spp.*, *B. minima*, *Ceramium spp.*, *E. turbinata* and *H. wrightii* can be considered typical of the North Sound area (Table 4), situated mostly in the mid-East portion of the lagoon. The green algae *Halimeda spp.* was present, although at different levels, in all sites of the South Bimini area, whereas in the North Sound only a sizable number of individuals were counted. *Laurencia spp*. was almost exclusive to the South Bimini area, being found only in three out of the nine sites in the North Sound area (Table 4, Figure 4).The distributions of most green algae species and sponges are highly correlated (coincident vectors in Figure 4) thus sharing common habitats, while the seagrass species and the red algae seem to be more widely distributed and colonize areas with different benthic and abiotic characteristics (non-coincident vectors in Figure 4).

### Seagrass analysis

#### Seagrass blade length and organic biomass

Statistically significant differences emerged between the values determined in the two study areas when comparing the average values of blade length for each seagrass species (Tables 5, 6 and 7).

**Table 5:** ***T. testudinum*** **and** ***H. wrightii*** **blade length values**

Species	Blade length (cm)	North Sound	South Bimini
*T. testudinum*	Mean ± SD	10.76 ± 5.36	14.48 ± 4.33
	Min	2.30	5.20
	Max	29.10	25.00
*H. wrightii*	Mean ± SD	5.25 ± 2.15	1.12 ± 1.72
	Min	1.10	1.40
	Max	12.10	5.60

**Table 6:** ***T. testudinum*** **and** ***H. wrightii*** **organic biomass in the nine North Sound sites**

Species	NS1	NS2	NS3	NS4	NS5	NS6	NS7	NS8	NS9
*T. testudinum*	0.78 g	0.99 g	1.49 g	1.86 g	1.00 g	1.52 g	1.57 g	2.64 g	1.29 g
*H. wrightii*	0.05 g	0.04 g	0.06 g	0.08 g	0.03 g	0.09 g	0.09 g	0.15 g	0.04 g

**Table 7:** ***T. testudinum*** **and** ***H. wrightii*** **organic biomass in the three South Bimini sites**

Species	SB1	SB2	SB3
*T. testudinum*	1.71 g	2.61 g	2.36 g
*H. wrightii*	0.00 g	0.04 g	0.00 g

On the average, both blade length and organic biomass for *T. testudinum* had values 1.5 times greater in South Bimini than in North Sound (p < 0.0001) (Tables 5, 6 and 7). There was substantial inter-site variability in the North Sound, possibly as a result of striking different local conditions of the abiotic factors.

On the other hand, *H. wrightii* leaves were longer in the North Sound (p < 0.0001) (Table 5) and the organic biomass resulted in a 16 fold higher value than in South Bimini (0.07 ± 0.04 g and 0.01 ± 0.02 g respectively) (Tables 6 and 7), an area with a very low occurrence of this seagrass, localized only in sites more distant from the shore. *H. wrightii* showed some inter-site variability, but to a lesser extent than for *T. testudinum*.

## Discussion

The North Sound and South Bimini are subtidal/intertidal habitats formed by sandy flats overlaying a Pleistocene pavement, dominated mainly by seagrass meadows interlaced with macroalgae. Both studied areas presented similarities in the benthic structure, and shared the presence of most of the species identified during this survey. However, given their morphological difference, one semi-closed and protected (North Sound), the other exposed to the open ocean (South Bimini), it is conceivable that two different habitats have developed as a consequence of interactions among several abiotic and biotic factors operating on different scale levels and in different time frames (Bell et al. 2006; Benedetti-Cecchi et al. 2003; Kornicker 1958). Moreover, the exposure to different levels of anthropogenic activities, with North Sound subjected to extensive alteration for the construction of a touristic complex, might have contributed to the differentiation between the underwater ecosystems of the two nursery grounds.

The analysis of the abiotic factors showed differences between the two areas (North Sound vs South Bimini) as well as variability among the nine sites in the North Sound, particularly between the Eastern side (still pristine as part of the Bimini MPA) and the Western side (close to the construction site), while a more uniform pattern was observed among the three sites in South Bimini. Such variations appear to be explainable at large by the natural conditions of the two areas, although the influence of human related activities over time should also be taken into consideration as possible contributing factor. Further analyses are required to investigate the possible impact of the anthropogenic activities carried out in the North Sound area on the local marine ecosystem.

The variability in sediment depth values recorded within both the North Sound and South Bimini can be explained by the presence of different bottom morphologies and constituents, and by the currents that continuously shift sediments (particularly the most fine-grained ones) along the sea floor. Unfortunately, during this study it was not possible to determine whether the different depth to pavement values across sites of the same area could be associated with the irregularity of the rocky basal pavement or to the irregularity of the sediment itself (mounds, dips), and a larger study would be needed to fully assess the factors responsible for the variability. With regards to South Bimini, the differing distance from shore of the three sites (the closest to shore, the greater accumulation due to more shelter and presence of mangrove roots that better retain sediment) and the constant sweeping of currents may also have contributed to the variability observed (Augustinus 1995; Carlton 1974).

Similarly, the natural differences in water currents and tidal flows existing between the protected North Sound lagoon and the exposed South Bimini coastline (Karleskint et al. 2010) could explain the significant difference in visibility between the two study areas, with North Sound showing substantially lower values, and the greater variation in salinity among the nine sites of the North Sound, compared to the more uniform pattern in South Bimini. However, clear differences in water clarity and greater variability in salinity coincided with the time period over which anthropogenic activities have increased in the North Sound area (Hussey 2003; Newman et al. 2007), and is a sign of concern. Consequently, the possible influence of constant fresh/drain water input from the construction site and nearby settlement on the North Sound cannot be excluded as a contributor to the differences observed between the two study areas and within the North Sound as well.

There were few differences in the distribution and abundance of macrobenthic epifauna or in species composition between the two areas, despite the different geomorphological structure and abiotic conditions. The variations that were detected were primarily related to broader distributions and the higher densities of most species in North Sound than South Bimini.

For most species, distribution and abundance varied within sites, particularly across the North Sound. Such differences are to be expected, given the presence of a range of biotopes (seagrass patches, sand flats and rocky bottom areas) influenced by the local morphological characteristics (e.g. bottom type, sediment composition and thickness) and variations in natural environmental factors, such as tidal flow, currents, and access to open ocean (Bell et al. 2006; Benedetti-Cecchi et al. 2003).

Overall seagrass and algal coverage were similar in the two study areas. However, although green algae distribution was relatively uniform in both areas, *T. testudinum* was more abundant in the clearer waters of South Bimini (mean density 2.5-fold higher than in the North Sound), while *H. wrightii* was primarily restricted to the more turbid North Sound. This matches with the idea that North Sound is more disturbed, with its central channel undergoing constant and frequent alterations in abiotic parameters due to currents and waves generated by boating, and increased variability in salinity. The increased disturbance regime in North Sound would benefit *H. wrightii* because it is tolerant to environmental fluctuations (Fong et al. 1997; McMillan 1974; Montague and Ley 1993) and chemical-physical and mechanical disturbances (e.g. dredging or boat passage) (Creed and Filho 1999). This seems to be the case, with patches of *H. wrightii* predominantly located in the disturbed central channel of North Sound. In contrast, *T. testudinum* is sensitive to disturbance, and although considered a stenohaline species, is intolerant to saline fluctuations (Moore 1963), which would explain its higher abundance in the more stable environment of South Bimini compared to the North Sound.

In terms of seagrass characteristics, *T. testudinum* blade length and blade density were substantially lower than those reported in the literature for the Caribbean area, although the habitat conditions appear comparable (seagrass flats parallel to shore, not far away and in shallow water 0.5–1 m). In fact, the average blade length in this study was 10–15 cm and average blade density was 50–150/m^2^, compared to published figures of 25–50 cm and 3000/m^2^, respectively (Brook 1978). These differences may however merely reflect local environmental conditions of Bimini. For *H. wrightii*, leaf length and organic biomass appeared quite uniform across all sites, but higher in the site located in the middle of the channel entrance of the North Sound, observations that are consistent with the density data.

At face value, both North Sound and South Bimini appear to still be in healthy condition, despite considerable anthropogenic activities, particularly on the Northern Island. The seagrass meadows are intermingled with macroalgae, and appear to be co-existing and not competing for resources (no take-over indicator signs were observed). In addition, both areas showed a degree of biodiversity and species abundance typical of such tropical underwater environments.

However, there are some disturbing patterns that suggest an early stage of ecosystem alteration. When comparing the results obtained in this study with those presented by Jennings et al. (2012), a loss in the biodiversity of the North Sound was observed: two species of seagrass *Syringodium filiforme* and *Halophila spp*., several macroalgae, porifera, arthropods and echinoderms whose presence in the lagoon was recorded in the survey performed in 2002 were not found in 2010. This change in species composition could be the result of either a gradual natural change/evolution of the local marine environment and/or as a long-term consequence of the anthropogenic activities taking place on the western side of the North Sound (pers. com. Gruber). In fact, if maintained, the presence of stressors in the North Sound has the potential to lead to gradual loss of the remaining seagrass species and seagrass-associated algae over the long term, and/or colonization by other species favoring the new habitat and conditions, with consequences for the entire ecosystem and its food webs. The difference in seagrass composition between the two areas, with the more environmentally sensitive *T. testudinum* (almost entirely confined to South Bimini, an area not directly exposed to human activity), appears to point in the same direction.

The data summarized in this study provide a set of baseline reference conditions to assist the monitoring future changes in the near-shore environments of Bimini’s shallow water nursery grounds. Periodic surveys of the area, including observations of the nektonic population and the status of the remaining mangrove forest, will allow early detection of environmental degradation and assist in preventing damage caused by the local anthropogenic development in the area, allowing for timely planning and preventive or corrective actions.

### Ethics statement

As the eastern half of the North Sound nursery ground is included in the Bimini Marine Protected Area created in 2000, in order to perform field measurements in such area a permit has been issued by the Bahamian Government.

All measurements have been carried out on public land and complying with local laws.

No protected species were sampled in this study.

## References

[CR1] Augustinus PGEF, Perillo GME (1995). Geomorphology and sedimentology of mangroves. Geomorphology and sedimentology of estuaries. Developments in sedimentology 53.

[CR2] Bell SS, Fonseca MS, Stafford NB, Larkum AWD (2006). Seagrass ecology: new contributions from a landscape perspective. Seagrasses: biology, ecology and conservation.

[CR3] Benedetti-Cecchi L, Bertocci I, Micheli F, Maggi E, Fosella T, Vaselli S (2003). Implications of spatial heterogeneity for management of marine protected areas (MPAs): examples from assemblages of rocky coasts in the northwest Mediterranean. Mar Environ Res.

[CR4] (2008). Bimini Bay Project Report (project 160191).

[CR5] Brook IM (1978). Comparative macrofaunal abundance in Turtlegrass (Thalassia testudinum) communities in South Florida characterized by high blade density. Bull Mar Sci.

[CR6] Carlton JM (1974). Land-building and stabilization by mangroves. Environ Conservat.

[CR7] Creed JC, Filho GMA (1999). Disturbance and recovery of the macroflora of a seagrass (Halodule wrightii Ascherson) meadow in the Abrolhos Marine National Park, Brazil: an experimental evaluation of anchor damage. J Exp Mar Biol Ecol.

[CR8] Feldheim KA, Chapman DD, Simpfendorfer CA, Richards VP, Shivji MS, Wiley TR, Sagarese S (2010). Genetic tools to support the conservation of the endangered smalltooth sawfish, Pristis pectinata. Conserv Genet Resour.

[CR9] Fong PM, Jacobson ME, Mesher MC, Lirman D, Harwell MC (1997). Investigating the management potential of a seagrass model through sensitivity analysis and experiments. Ecol Appl.

[CR10] Hedgpeth JW (1957). Classification of marine environments. Mem Geol Soc Am.

[CR11] Hussey N (2003). An evaluation of Landsat 7 ETM + satellite imagery for quantitative biotope mapping of the Bimini islands, the Bahamas including two known lemon shark (Negaprion brevirostris) nursery grounds. Master thesis.

[CR12] Jennings DE, Gruber SH, Franks BR, Kessel ST, Robertson AL (2008). Effects of large-scale anthropogenic development on juvenile lemon sharks (Negaprion brevirostris) populations. Environ Biol Fishes.

[CR13] Jennings DE, Di Battista JD, Stump KL, Hussey NE, Franks BR, Grubbs RD, Gruber SH (2012). Assessment of the aquatic biodiversity of a threatened coastal lagoon at Bimini, Bahamas. J Coast Conservat.

[CR14] Karleskint G, Turner R, Small J (2010). Introduction to marine biology, Cengage Learning.

[CR15] Kenneth WA (2005). A re-examination of fish estuarine dependence: evidence for connectivity between estuarine and ocean habitats. Estuar Coast Shelf Sci.

[CR16] Kornicker LS (1958). Ecology and taxonomy of recent marine ostracodes in the Bimini area, Great Bahama Bank. Texas University, Institute of Marine Science, vol. 5.

[CR17] Marbà N, Gallegos ME, Merino M, Duarte CM (1994). Vertical growth of Thalassia testudinum: seasonal and interannual variability. Aquat Bot.

[CR18] McMillan C, Reimold RJ, Queen WH (1974). Salt tolerance of mangroves and submerged aquatic plants. Ecology of halophytes.

[CR19] Montague CL, Ley JA (1993). A possible effect of salinity fluctuation on abundance of benthic vegetation and associated fauna in Northern Florida Bay. Estuaries.

[CR20] Moore DR (1963). Distribution of the seagrass, Thalassia, in the United States. Bull Mar Sci Gulf Caribb.

[CR21] Morrisey JF, Gruber SH (1993). Home range of Juvenile Lemon Sharks, Negaprion brevirostris. Copeia.

[CR22] Murchie KJ, Schwager E, Cooke SJ, Danylchuk AJ, Danylchuk SE, Goldberg TL, Suski CD, Philipp DP (2010). Spatial ecology of juvenile lemon sharks (Negaprion brevirostris) in tidal creeks and coastal waters of Eleuthera, The Bahamas. Environ Biol Fishes.

[CR23] Nagelkerken I, Roberts CM, Van der Velde G, Dorenbosch M, Van Riel MC, Cocheret de la Morinière E, Nienhuis PH (2002). How important are mangroves and seagrass beds for coral reef fish? The nursery hypothesis tested on an island scale. Mar Ecol Progr.

[CR24] Newman SP, Handy RD, Gruber SH (2007). Spatial and temporal variations in mangrove and seagrass faunal communities at Bimini, Bahamas. Bull Mar Sci.

[CR25] Orth RJ, Carruthers TJB, Short FT, Dennison WC, Duarte CM, Fourqurean JW, Williams SL (2006). A global crisis for seagrass ecosystems. Bioscience.

[CR26] Parrish JD (1989). Fish communities of interacting shallow-water habitats in tropical oceanic regions. Mar Ecol Prog Ser.

[CR27] Preisendorfer RW (1986). Secchi disk science: visual optics of natural waters. Limnol Oceanogr.

[CR28] Rönnback P (1999). The ecological basis for economic value of seafood production supported by mangrove ecosystem. Ecol Econ.

[CR29] Scoffin TP (1970). The trapping and binding of subtidal carbonate sediments by marine vegetation in Bimini lagoon, Bahamas. J Sediment Res.

[CR30] Sealey KS (2004). Large-scale ecological impacts of development on tropical islands systems: comparison of developed and undeveloped islands in the central Bahamas. Bull Mar Sci.

[CR31] Stoner W (1980). The role of seagrass biomass in the organization of benthic macrofaunal assemblages. Bull Mar Sci.

[CR32] Turekian KK (1957). Salinity variations in sea water in the vicinity of Bimini, Bahamas, British West Indies.

[CR33] Voss GL, Voss NA (1960). An ecological survey of the marine invertebrates of Bimini, Bahamas, with a consideration of the zoogeographical relationships. Bull Mar Sci Gulf Caribb.

